# Clinical and Molecular Profile of Patients With Condyloma Acuminatum Treated in the Brazilian Public Healthcare System

**DOI:** 10.7759/cureus.21961

**Published:** 2022-02-06

**Authors:** Fernando Nestor Facio, Maria Fernanda Warick Facio, Ana Clara Nagle Spessoto, Moacir Godoy, Hariel Tessaro, Ricardo Campos, Diego Zanatto, Marilia Calmon, Paula Rahal, Luís Cesar Fava Spessoto

**Affiliations:** 1 Urology, Faculty of Medicine of São José do Rio Preto (FAMERP), São José do Rio Preto, BRA; 2 Medicine, Faceres Medical School, São José do Rio Preto, BRA; 3 Medicine, Medical School of Catanduva, Catanduva, BRA; 4 Cardiology, Faculty of Medicine of São José do Rio Preto (FAMERP), São José do Rio Preto, BRA; 5 Biology, Universidade Estadual Paulista (UNESP), São José do Rio Preto, BRA

**Keywords:** sexually transmitted diseases, warts, hpv, human papillomavirus, condyloma

## Abstract

Condyloma acuminatum is a common clinical outcome of human papillomavirus (HPV) in men. A prospective investigation was performed of the clinical and molecular profile of 122 patients with condyloma acuminatum treated by the Brazilian public healthcare system. The patients were evaluated clinically. The fragments were submitted to molecular analysis for the identification of HPV genotypes. A total of 104 (85.2%) patients presented multiple warts and 18 (14.8%) presented a single wart. The predominant location was the body of the penis (48.4% of cases of multiple warts and 7.4% of cases of single warts), and 49 (40.2%) cases were recurrences and 73 (59.8%) were initial occurrences. Regarding sexual activity, 56 patients (45.9%) had multiple partners and 65 (53.3%) had a single partner. The most frequent genotype was HPV6 (70%). In conclusion, the most frequent anatomic location of condyloma acuminatum was the body of the penis in the present sample. The present findings suggest that the natural history of infection by HPV is not yet completely known and that greater care is needed to ensure clinical safety in the follow-up of these patients due to the oncogenic potential.

## Introduction

Condyloma acuminatum, also known as venereal warts, is caused by the human papillomavirus (HPV) [1]. HPVs are small, non-enveloped, epitheliotropic viruses that have circular double-stranded DNA with approximately 8,000 base pairs. These viruses are responsible for subclinical disease and are associated with premalignant lesions and some intraepithelial neoplasms [2]. There are more than 200 known genotypes of HPV, which are responsible for both benign and malignant tumors [3]. Nearly 40 types infect the anogenital tract and are among those considered high of oncogenic risk [3]. High-risk HPVs are transmitted sexually; sexual activity at an early age and having a large number of sexual partners, along with smoking, oral contraceptives, and other sexually transmitted diseases, are factors that contribute to an increased risk of infection by HPV [2]. Among high-risk types of HPV, HPV16 is the most frequently detected on the population level, causing invasive cervical cancer in approximately 60% of patients, followed by HPV18 (in approximately 15%) [4-7]. The genotypes most commonly detected in condyloma are HPV6 and 11 (96 to 100% of all cases) [8,9]. Condyloma is a common clinical outcome of infection by HPV in men, especially those between 25 and 29 years of age [10]. Non-oncogenic HPV6 and HPV11 are the etiological agents responsible for more than 90% of cases of condylomatous lesions [11]. However, information on the incidence of HPV and condyloma in Brazilians is limited to a small number of studies [9,12,13]. Knowledge regarding types of HPV detected in individuals with condyloma, infection rate, and progression of the disease is clinically relevant and could contribute considerably to the development of public prevention and treatment policies in Brazil. Therefore, the aim of the present study was to investigate the clinical and molecular profiles of patients with condyloma acuminatum in the Brazilian public healthcare system.

## Materials and methods

Study material

A prospective investigation was performed, which included 122 male patients with condyloma acuminatum, regardless of race, under the care of urologists of the public healthcare system in a city in the state of São Paulo, Brazil. Patients younger than 18 years of age and those who did not agree to participate in the study were excluded. This study received approval from the local Institutional Review Board (certificate number: 89039318.3.0000.5415).

The patients were assessed clinically considering age, signs and symptoms, location, time of appearance and evolution of the lesion, sexual activity (multiple partners), and vaccination for HPVs. All patients were subjected to surgical treatment under local anesthesia. Postoperative complications were not found at the outpatient return two weeks after the procedure. The fragments removed during the surgical procedure were sent to the Pathology and Genomic Studies Laboratories of the Institute of Biosciences, Letters, and Exact Sciences of Universidade Estadual Paulista (UNESP).

DNA was extracted from the samples using the phenol-chloroform method. Initially, 200 μL of TEP solution (proteinase K 1 mg/mL; SDS 1%, tris HCl 10 mM, pH 8.0; EDTA 1 mM pH 8.0) was added to 200 μL of sample and the mixture was shaken at 37ºC overnight, followed by incubation at 56ºC for one hour. Next, 200 μL of a phenol:chloroform:isoamyl alcohol solution (25:24:1) was added at 4ºC, followed by homogenization for 1 minute and centrifugation at 12,000 rpm for 5 minutes at room temperature. For DNA precipitation and washing, DNA, 500 μL of 100% ethanol, and 20 μL of ammonium acetate 3M was added and the microtubes were incubated for two hours at -80°C, followed by centrifugation at 12,000 rpm for 20 minutes at 4ºC, with the subsequent discarding of the aqueous phase. Also, 500 μL of 75% ethanol was added to the precipitate at -20ºC, followed by centrifugation at 12,000 rpm for 10 minutes at 4ºC and discarding of the aqueous phase. The presence and integrity of DNA from the samples were determined using polymerase chain reaction (PCR) with the oligonucleotide primers GSTP1 - F (5’GTAGTTTGCCCAAGGTCAAG3’) and GSTP1 - R (5’AGCCACCTGAGGGGTAAG3’) [14]. The amplification of the L1 region of the HPV was conducted in two steps (nested PCR). The first step was PCR using the oligonucleotide primers My09 (5’CGTCCMARRGGAWACTGATC3’) and My11 (5’GCMCAGGGWCATAAYAATGG3’) [15] with a final volume of 25 μL containing 10x buffer, 1.0 μM of dNTP (Applied Biosystems, Foster City, CA, USA), 0.4 μM of My09, 0.4 μM of My11, 5U of Taq DNA polymerase (Sinapse Inc., São Paulo, Brazil), Milli-Q H2O, and 2.5 μL of DNA. The second step of the nested PCR for the amplification of HPV was performed with the oligonucleotide primers GP5 (5’TTTGTTACTGTGGTAGATAC3’) and GP6 (5’GAAAAATAAACTGTAAATCA3’) [16] to amplify an internal sequence composed of 150 bp. The amplification products of the GP5/GP6 oligonucleotides were purified using the DNA Clean & ConcentratorTM kit (ZYMO, Irvine, CA, USA) following the manufacturer’s instructions. The purified product was eluted in 10 μL of Milli-Q water and stored at -20ºC.

The purified product was sequenced using the Sanger method [16] with the aid of the BigDye Terminator kit (Applied Biosystems/Life Technologies, Foster City, CA, USA).

Statistical analysis

Data analysis involved descriptive and inferential statistics. Continuous quantitative variables with Gaussian distribution were expressed as mean, standard deviation, and median values. Categorical variables were expressed as quantity and relative frequency. The data were analyzed using Fisher’s exact test. All analyses were performed in the StatsDirect Statistical Software (version 3.3.4, 2020). The acceptable alpha error rate was set at 5%, and a p-value of ≤0.05 was considered indicative of statistical significance.

## Results

A total of 122 men were evaluated. Age ranged between 18 and 79 years (mean: 34.3 ± 11.3 years; median: 31 years). Regarding marital status, 64 (52.4%) were single, 47 (38.5%) were married, and 11 (9.0%) were in other categories. Most (84 cases; 68.8%) were residents of the city of São José do Rio Preto, Sao Paulo, and the other 31.2% resided in neighboring cities. A total of 17.2% of the patients were students. In the clinical analysis, 104 patients (85.2%) had multiple warts and 18 (14.8%) had a single wart. The predominant location was the body of the penis (48.4% of cases of multiple warts and 7.4% of cases of single warts) (Table 1).

**Table 1:** Distribution of warts in the genital region Values in parentheses indicate the percentage.

Warts	Body of the penis	Pubis	Glans	Scrotum	Total
Multiple	59 (48.4)	24 (19.7)	20 (16.4)	1 (0.8)	104
Single	9 (7.4)	5 (4.1)	4 (3.3)	0 (0)	18
Total	68	29	24	1	122

The results of the distribution of initial and recurrent warts, sexual partners and form of presentation of warts, sexual partners and clinical evolution, and presentation of warts and HPV6 and HPV11 genotypes in patients with condyloma acuminatum are expressed in Table 2; 49 (40.2%) cases were recurrences and 73 (59.8%) were initial occurrences. No statistically significant differences were found between initial or recurring warts in the patients with multiple and single presentations (p = 0.7959; Fisher’s exact test). In terms of sexual activity, 56 (45.9%) patients reported having multiple partners and 65 (53.3%) reported having only one partner. No significant difference was found between patients with multiple or single partners regarding initial or recurring warts (p = 0.3077; Fisher’s exact test). No significant association was found between the number of sexual partners and clinical evolution (primary or recurring) (p = 0.7112; Fisher’s exact test). Fisher’s exact test revealed no statistically significant association between single or multiple warts and the most prevalent types of HPV (HPV6 x HPV11; p = 0.2369).

**Table 2:** Distribution of initial and recurrent warts, sexual partners and form of presentation of warts, sexual partners and clinical evolution, and presentation of warts and HPV6 and HPV11 genotypes in patients with condyloma acuminatum by the Fisher’s exact test. HPV, human papillomavirus

Warts/Presentation	Initial	Recurrent	p-Value
Multiple	63	41	0.7959
Single	10	8	
Partners/presentation	Multiple	Single	p-Value
Multiple	50	6	0.3077
Single	53	12	
Partners/evolution	Initial	Recurring	p-Value
Multiple	35	21	0.7112
Single	38	27	
Warts	HPV6	HPV11	p-Value
Single	8	5	0.2369
Multiple	63	19	

The vaccination rate was very low. Among the 122 cases, only three (2.4%) received vaccinations (bivalent vaccine). Regarding the molecular analysis of the fragments collected during the surgical procedure, genotyping was performed on the material of 102 patients (Table 3).

**Table 3:** Genotypes identified in fragments from patients with condyloma acuminatum HPV, human papillomavirus

Genotype	n	% Ssubtypes
HPV6	71	69.6
HPV11	25	24.5
HPV16	1	1.0
HPV18	3	2.9
HPV40	1	1.0
HPV44	1	1.0

The main location of warts according to HPV6 and HPV11 genotypes was body of the penis (40 and 13, respectively). The comparative analysis using the chi-square test revealed no statistically significant difference regarding the location of the warts (glans, body of the penis, or pubis) for the two most prevalent types of HPV (HPV6 x HPV11; p = 0.1631).

## Discussion

The present results show that patients with condyloma acuminatum are young adults and students with multiple warts located predominantly on the body of the penis, with HPV6 as the most frequent genotype. The prevalence of HPV in men ranges from 1.3% to 72.9%, depending on the type of sample and detection method. However, the majority of studies have been conducted with specific populations [17,18]. In the present investigation, the sample was composed of individuals from the general population.

As knowledge on the natural history of HPV is limited to information from infection in men, the criteria need to be broadened in order to detect the presence of one or more genotypes on the penis and in the seminal fluid with high specificity [19]. There is no diagnostic test for HPV in men. The challenge is to establish the relationship of partners with HPV lesions and the incidence of penile and cervical cancer. Although three DNA kits for HPV are commercially available for women (Hybrid Capture II [Digene Corporation, Gaithersburg, MD, USA], Cervista HPV HR and Cervista HPV 16/18 [Hologic Inc, Bedford, Bedford, MA, USA]), the Food and Drug Administration has not yet approved any tests for men [20].

An important consideration for the detection of HPV in men is the anatomic location. Indeed, there is considerable variability in the incidence of the virus depending on the location (penile body, glans, foreskin, coronal sulcus); samples from the urethra and semen have less positivity for the detection of HPV [21]. There are currently more than 200 genotypes identified, two of which (6 and 11) are associated with condyloma and four (16, 18, 31, and 33) are associated with cancer [22].

A cohort study demonstrated that high-risk HPV genotypes have a lower viral depuration rate in non-vaccinated men (p < 0.001). Infection by high-risk HPVs has a greater probability of persisting over time compared to other genotypes [23]. The detection of HPV in men should be considered a high priority, as the male population acts as a vector and reservoir of this virus. As HPV is present in anal, oral, and penial neoplasms, it is important to conduct population-based studies on the prevalence of HPV in men. For such, the use of condyloma samples from different anatomic areas of the penis could broaden knowledge in this field.

Limitations

This work is based on a prospective study performed at a single medical center. Long-term and multi-centric studies are necessary to confirm the oncogenic potential of patients infected by HPV.

## Conclusions

In the present study, the most frequent anatomic location of condyloma acuminatum in the present sample was the body of the penis. The present findings suggest that the natural history of infection by HPV is not yet completely known in men. However, urologists should improve their knowledge regarding HPV to ensure clinical safety in the follow-up of these patients due to the oncogenic potential.
